# The Cultural Evolution and Social Pressures of Liposuction Among Iraqi Women: Navigating Modern Beauty Standards Amidst Conservative Traditions

**DOI:** 10.7759/cureus.61440

**Published:** 2024-05-31

**Authors:** Ahmed R Al-Aibi, Mustafa Ismail

**Affiliations:** 1 Department of Surgery, Baghdad Teaching Hospital, Medical City, Baghdad, IRQ; 2 Department of Neurosurgery, College of Medicine, University of Baghdad, Baghdad, IRQ

**Keywords:** iraq, cosmetic procedures, beauty standards, cultural expectations, societal norms

## Abstract

This editorial explores the impact of societal transformation on Iraqi women's lives, particularly concerning beauty standards and cosmetic procedures like liposuction. It examines the influences of modernization, social media, and social pressures juxtaposed with traditional conservative values. The transition from repressive regimes to more open, albeit unstable, political landscapes has led to significant shifts in women's roles, with increased conservatism contrasting with emerging modernist influences. The role of social media in amplifying modern beauty ideals creates internal conflicts for women striving to balance these with traditional expectations. The growing acceptance of cosmetic procedures indicates a shift toward integrating modern beauty standards within Iraqi society.

## Editorial

Introduction

The transformation of societal norms and cultural expectations in Iraq has significantly impacted women's lives, particularly in relation to beauty standards and cosmetic procedures like liposuction. Over the past few decades, Iraq has experienced substantial political and social upheavals, leading to shifts in gender roles and expectations. As a result, women in Iraq are increasingly influenced by global beauty trends and social media, which often present ideals that conflict with deeply rooted conservative values. This editorial explores the complex interplay of modernization, social media, and social pressures on Iraqi women, highlighting the tensions between embracing contemporary beauty standards and adhering to traditional cultural norms. Through examining these influences, we aim to understand the broader implications for women's identities and choices in a rapidly changing society [1].

Cultural changes and modernization

The role of women in Iraq has undergone substantial changes over the past few decades, influenced by various political and social upheavals. During the Ba'athist regime and subsequent conflicts, women's roles were highly contested and shaped by state policies and economic conditions. The transition from a repressive regime to a more open, albeit unstable, political landscape has seen shifts toward both greater conservatism and modernization. The economic sanctions and conflicts of the 1990s and early 2000s also contributed to these shifts, with increased conservatism contrasting with emerging modernist influences [2,3].

Social media effects

Social media has played a crucial role in transforming beauty standards among Iraqi women. Platforms like Instagram and Facebook expose women to global beauty trends, increasing the desire for cosmetic procedures such as liposuction. This exposure often leads to internal conflicts between modern beauty ideals and traditional values. The influence of social media is evident in the increasing number of women seeking cosmetic enhancements to align with perceived international standards of beauty. Platforms like Instagram and Facebook expose women to images of idealized beauty, leading to internal conflicts and a stronger desire for cosmetic procedures such as liposuction. Studies have shown that women who frequently use social media and follow beauty influencers are more likely to consider cosmetic surgery to achieve these beauty ideals [4,5].

Social pressure and conservative culture

Iraqi society remains deeply rooted in conservative traditions, which often conflict with the aspirations fostered by modernization and social media. Women face substantial social pressure to conform to traditional roles while simultaneously being influenced by modern beauty standards. This dichotomy creates a unique tension where women are torn between adhering to conservative expectations and pursuing contemporary beauty ideals.

Historically, Iraqi society has been characterized by its conservative social fabric, where women's roles have been largely confined to domestic spheres. Despite the long-standing conservative atmosphere, there have been notable Iraqi women who have made significant contributions in various fields. The decline of the Ottoman Empire and the subsequent formation of the Iraqi state under colonial influences further entrenched conservative values, impacting women's roles and rights. These conservative norms have persisted, creating ongoing social pressures for women to adhere to traditional roles even as they navigate modern influences [1]. During the Ba'athist regime and subsequent conflicts, women's roles were highly contested and shaped by state policies and economic conditions. The transition from a repressive regime to a more open, albeit unstable, political landscape has seen shifts towards both greater conservatism and modernization [2].

The period of economic sanctions in the 1990s and the subsequent rise of Islamist political parties have reinforced social conservatism. This era saw a drastic shift toward greater social conservatism, which continues to influence gender ideologies and relations. Despite these conservative pressures, women have increasingly sought to redefine their roles within society, often facing significant resistance [2,3].

The rapid modernization and exposure to global beauty standards through social media have amplified the tension between traditional roles and contemporary aspirations. Women are under immense pressure to conform to traditional expectations while also being influenced by modern beauty ideals propagated by social media. This has led to a growing interest in cosmetic procedures like liposuction, as women strive to meet these conflicting standards. Social media platforms such as Instagram and Facebook expose women to global beauty trends, increasing the desire for cosmetic enhancements. This exposure often leads to internal conflicts between modern beauty ideals and traditional values [4]. The influence of social media is evident in the increasing number of women seeking cosmetic enhancements to align with perceived international standards of beauty.

The influence of social media has been particularly significant in urban areas, where the exposure to Western beauty standards is more pronounced. Women in these areas are often caught between the conservative expectations of their families and communities and the desire to emulate the glamorous lifestyles and appearances they see online. This tension can lead to psychological stress and a sense of inadequacy as women struggle to reconcile these opposing pressures. The pursuit of cosmetic procedures, such as liposuction, becomes a way for these women to navigate this complex social landscape, attempting to balance traditional values with modern aspirations.

Additionally, the influence of conservative religious leaders and traditional cultural norms continues to play a significant role in shaping societal expectations. Many women face backlash or criticism for pursuing cosmetic enhancements, which are often seen as contrary to traditional values of modesty and natural beauty. This societal pressure can lead to secrecy and stigma surrounding cosmetic procedures, further complicating the decision-making process for women considering these options. Despite these challenges, the growing accessibility and popularity of cosmetic procedures indicate a shift toward greater acceptance and integration of modern beauty standards within Iraqi society.

Conclusion

The evolving cultural landscape in Iraq presents a complex interplay between modernization, social media influences, and traditional values. As Iraqi women navigate these dynamics, it is essential to foster an environment that supports informed choices and balances modern aspirations with cultural heritage. Encouraging open dialog and education about cosmetic procedures like liposuction can help mitigate the risks and promote healthier decisions.
